# Supplementary material for: A quantitative geospatial analysis of the risk that Boko Haram will target a school
Source: PLoS One. 2025 Jun 17;20(6):e0320939. doi: 10.1371/journal.pone.0320939 (PMC12173403; doi:10.1371/journal.pone.0320939)
Supplement: S1 Appendix A — (PDF) [file pone.0320939.s001.pdf]

Supplementary Material

Appendix A: List of Features Associated with Each School

Independent Feature	Feature Definition
1st_closest_distance_km	Distance (km) to the 1st closest security installation from the school
2nd_closest_distance_km	Distance (km) to the 2nd closest security installation from the school
3rd_closest_distance_km	Distance (km) to the 3rd closest security installation from the school
4th_closest_distance_km	Distance (km) to the 4th closest security installation from the school
5th_closest_distance_km	Distance (km) to the 5th closest security installation from the school
5km_radius_total_attacks	Total number of attacks within a 5 km radius of the school
10km_radius_total_attacks	Total number of attacks within a 10 km radius of the school
25km_radius_total_attacks	Total number of attacks within a 25 km radius of the school
50km_radius_total_attacks	Total number of attacks within a 50 km radius of the school
Is_Location_Rural	Binary indicator for rural location
Is_Location_Urban_Centre	Binary indicator for urban center location
Is_Location_Urban_Cluster	Binary indicator for urban cluster location
nga_commun	Communication Risk Score: Avg. risk score (1-5) for communication access at the school level based on ward's communication access risk
nga_exposu	Exposure Risk Score: Avg. risk score (1-5) at the ward level, considering factors like population density, household proximity, and hygiene indicators
nga_socioe	Socioeconomic Risk Score: Avg. socioeconomic vulnerability risk score (1-5) for each school at the ward level

Table 9. Independent features calculated for every school record and their description.
