# Supplementary material for: A quantitative geospatial analysis of the risk that Boko Haram will target a school
Source: PLoS One. 2025 Jun 17;20(6):e0320939. doi: 10.1371/journal.pone.0320939 (PMC12173403; doi:10.1371/journal.pone.0320939)
Supplement: S2 Appendix B — (PDF) [file pone.0320939.s002.pdf]

Appendix B: Performance of All Machine Learning Classifiers

DV_k	**Classifier**	**Precision**	**Recall**	**F1-Score**	**AUC**
1 km.	Random Forest	0.93	0.19	0.31	0.992
1 km.	Decision Tree	0.85	0.25	0.38	0.987
1 km.	AdaBoost	0.83	0.62	0.71	0.81
1 km.	Logistic Regression	0.00	0.00	0.00	0.909
1 km.	Linear SVM	0.00	0.00	0.00	0.923
1 km.	Gaussian NB	0.07	0.45	0.13	0.936
1 km.	MLP	0.38	0.13	0.19	0.904
1 km.	DNN	0.00	0.00	0.00	0.981
2 km.	Random Forest	0.85	0.63	0.72	0.998
2 km.	Decision Tree	0.8	0.79	0.79	0.99
2 km.	AdaBoost	0.92	0.91	0.91	0.95
2 km.	Logistic Regression	0.67	0.11	0.19	0.902
2 km.	Linear SVM	0.6	0.11	0.19	0.933
2 km.	Gaussian NB	0.22	0.62	0.32	0.954
2 km.	MLP	0.74	0.23	0.35	0.984
2 km.	DNN	0.67	0.28	0.39	0.995
3 km.	Random Forest	0.9	0.71	0.8	0.994
3 km.	Decision Tree	0.86	0.84	0.85	0.984
3 km.	AdaBoost	0.94	0.92	0.93	0.96
3 km.	Logistic Regression	0.75	0.44	0.56	0.921
3 km.	Linear SVM	0.83	0.1	0.18	0.94
3 km.	Gaussian NB	0.34	0.7	0.45	0.956
3 km.	MLP	0.76	0.74	0.75	0.991
3 km.	DNN	0.76	0.83	0.80	0.998
5 km.	Random Forest	0.95	0.73	0.82	0.996
5 km.	Decision Tree	0.94	0.67	0.78	0.988
5 km.	AdaBoost	0.96	0.97	0.97	0.98
5 km.	Logistic Regression	0.95	0.48	0.61	0.957
5 km.	Linear SVM	0.96	0.44	0.61	0.961
5 km.	Gaussian NB	0.35	0.72	0.47	0.958
5 km.	MLP	0.88	0.78	0.83	0.995
5 km.	DNN	0.82	0.92	0.87	0.998
10 km.	Random Forest	0.99	0.63	0.77	0.91
10 km.	Decision Tree	0.98	0.68	0.81	0.979
10 km.	AdaBoost	0.99	0.98	0.98	0.99
10 km.	Logistic Regression	0.96	0.48	0.64	0.937
10 km.	Linear SVM	0.96	0.48	0.64	0.951
10 km.	Gaussian NB	0.43	0.61	0.51	0.937
10 km.	MLP	0.92	0.85	0.88	0.993
10 km.	DNN	0.96	0.88	0.92	0.998

Table 10. Performance Metrics of Various Classifiers Across Different Dataset Variations
