# Supplementary material for: A quantitative geospatial analysis of the risk that Boko Haram will target a school
Source: PLoS One. 2025 Jun 17;20(6):e0320939. doi: 10.1371/journal.pone.0320939 (PMC12173403; doi:10.1371/journal.pone.0320939)
Supplement: S3 Appendix C — (PDF) [file pone.0320939.s003.pdf]

Appendix C: Decision Tree Graphs

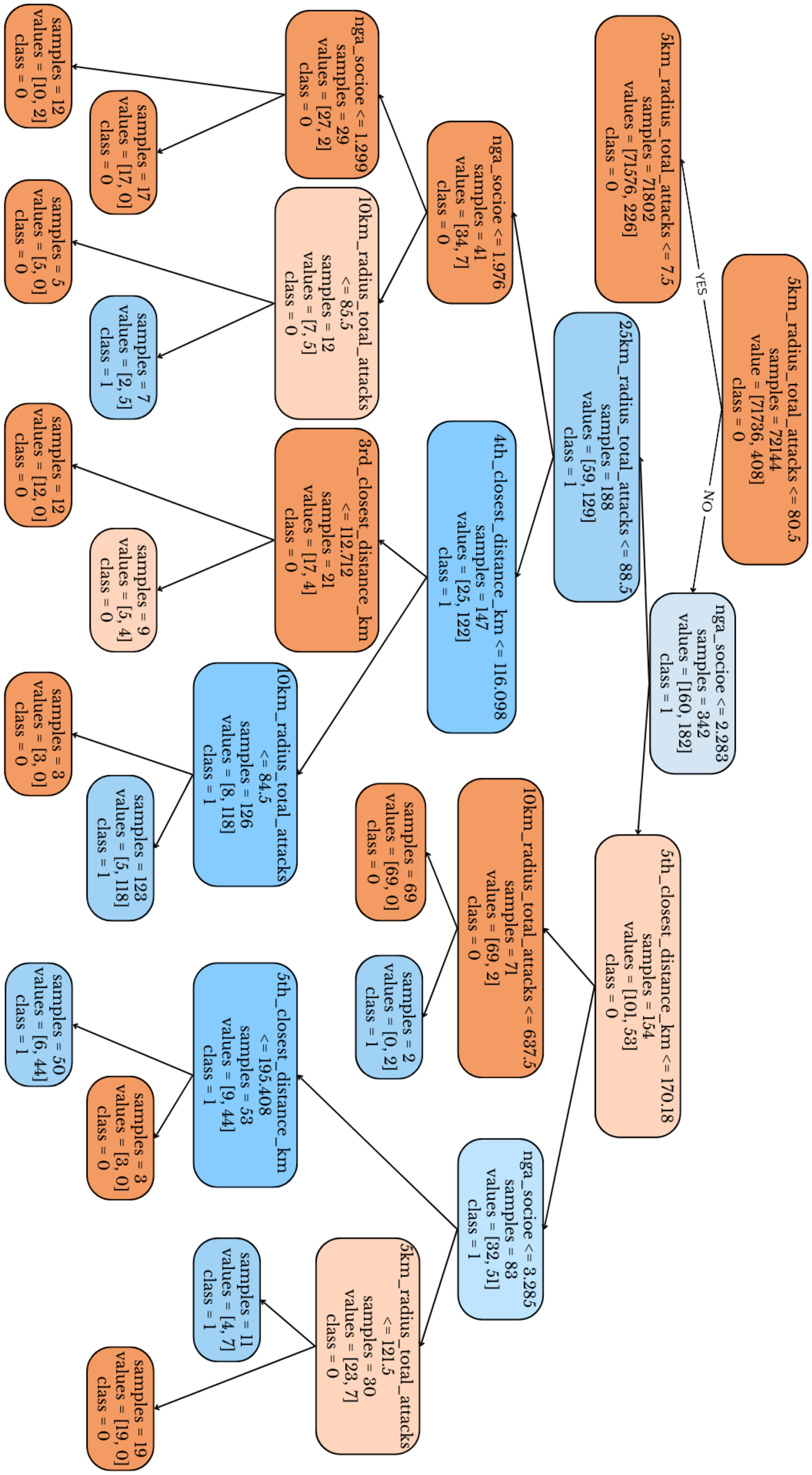

Fig 16. Relevant Part of the Decision tree extracted for the $k = 2$ Dependent Variable.

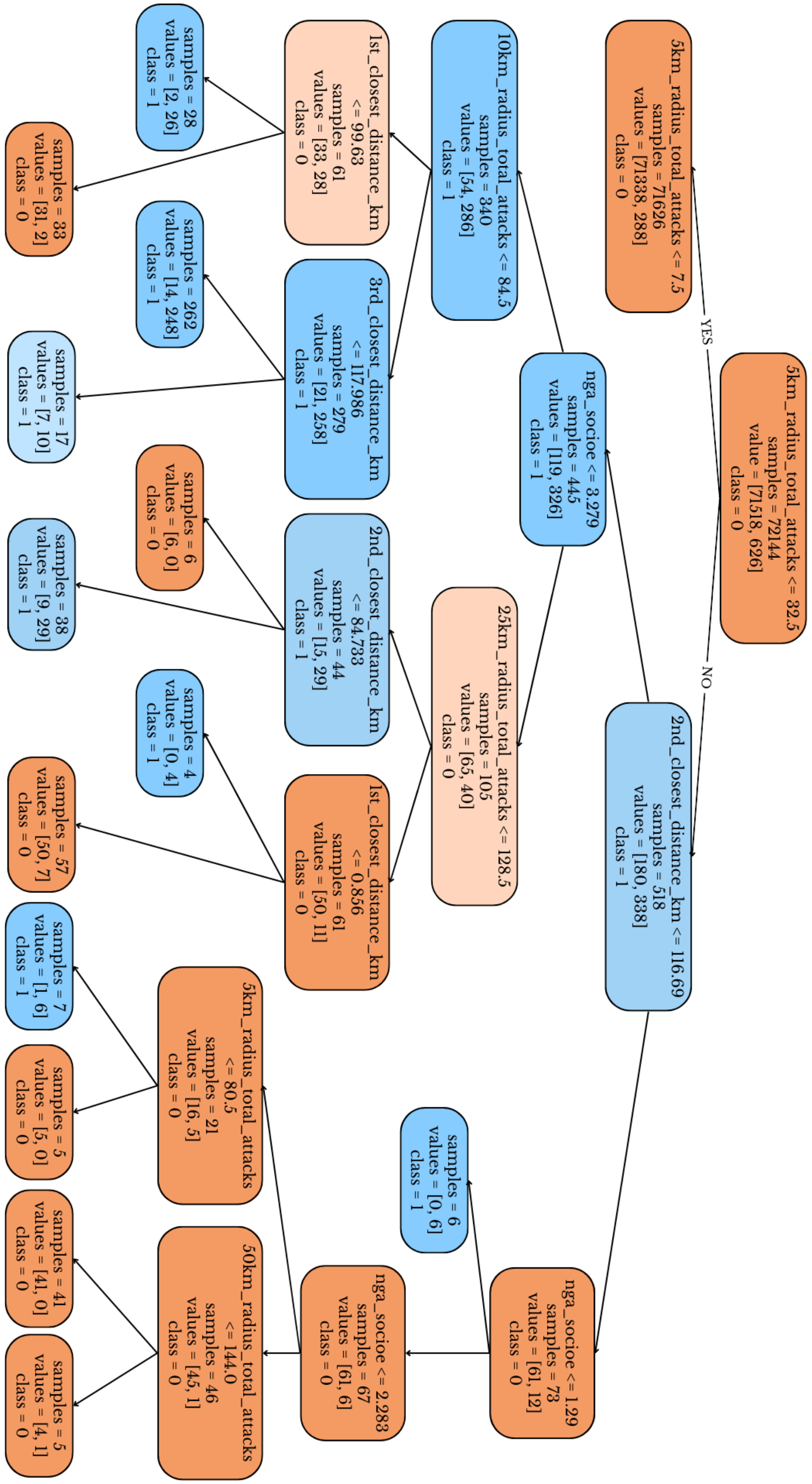

Fig 17. Decision tree extracted for the $k = 3$ Dependent Variable.

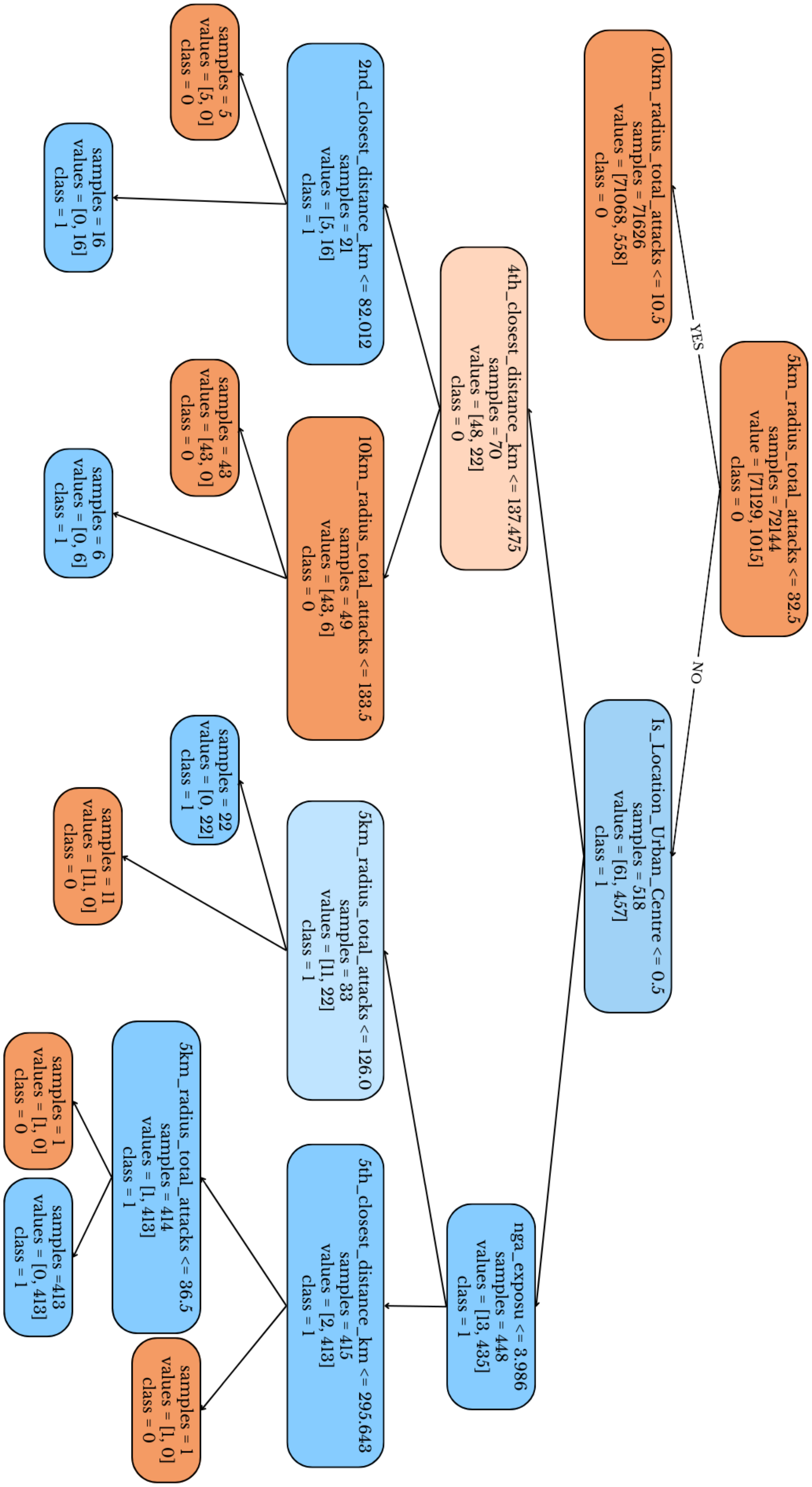

Fig 18. Relevant Part of the Decision tree extracted for the $k = 5$ Dependent Variable.

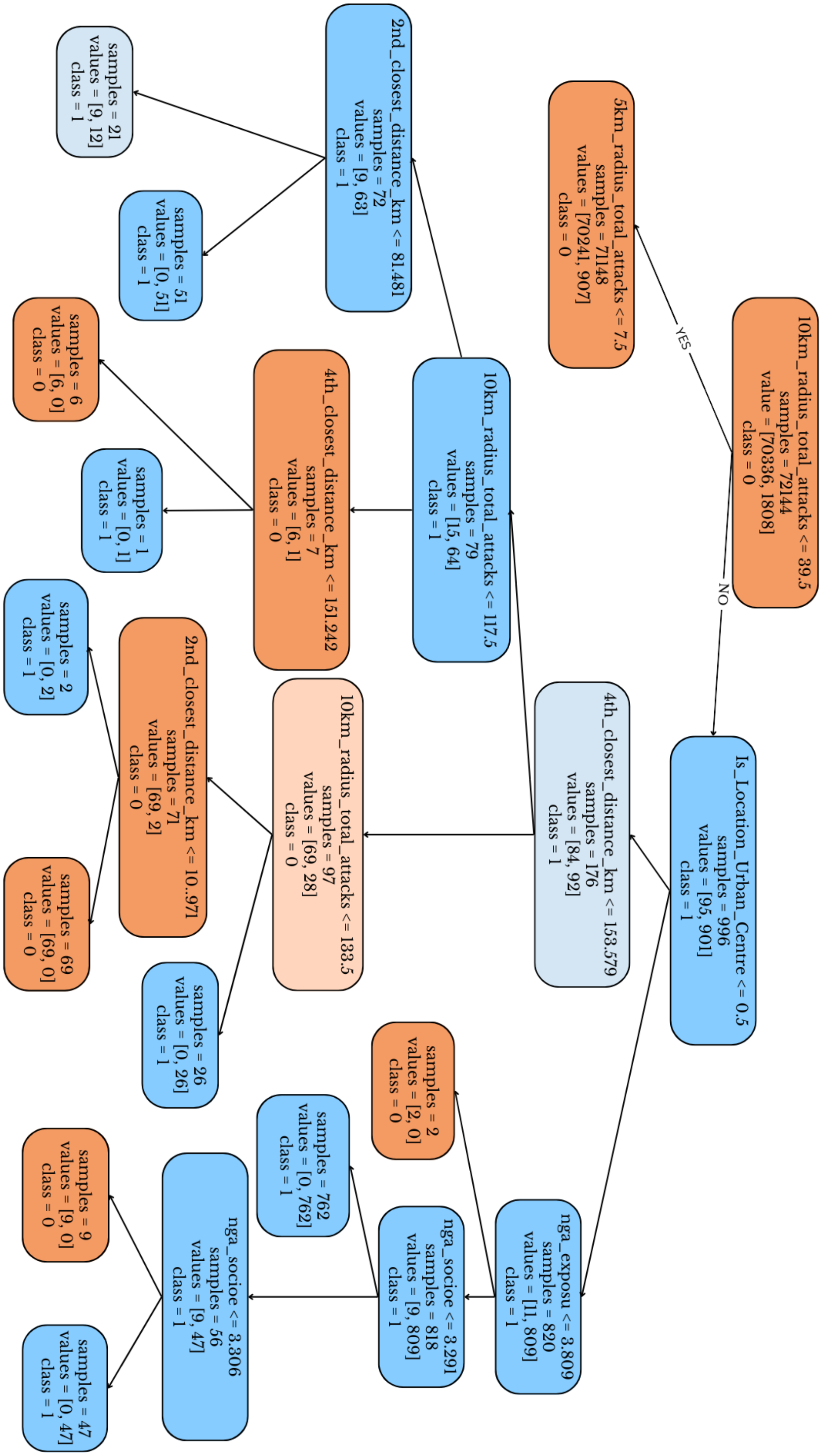

Fig 19. Relevant Part of the Decision tree extracted for the $k = 10$ Dependent Variable.
