# Supplementary material for: A quantitative geospatial analysis of the risk that Boko Haram will target a school
Source: PLoS One. 2025 Jun 17;20(6):e0320939. doi: 10.1371/journal.pone.0320939 (PMC12173403; doi:10.1371/journal.pone.0320939)
Supplement: S4 Appendix D — (PDF) [file pone.0320939.s004.pdf]

Appendix D: Ward's Wealth and Residential Area Type

**Risk Score**	**Rural**	**Urban Centre**	**Urban Cluster**
Communication 1 to 2	33.65%	96.65%	44.96%
Communication 2 to 3	25.37%	3.17%	30.83%
Communication 3 to 4	21.72%	0.17%	17.62%
Communication 4 to 5	19.26%	0.06%	6.58%
Exposure 1 to 2	0.0035%	0%	0%
Exposure 2 to 3	5.5%	1.03%	2.29%
Exposure 3 to 4	75.29%	56.54%	71.74%
Exposure 4 to 5	19.21%	42.43%	25.97%
Socioeconomic 1 to 2	2.03%	65.49%	4.5%
Socioeconomic 2 to 3	12.59%	26.22%	25.21%
Socioeconomic 3 to 4	30.19%	7.42%	26.26%
Socioeconomic 4 to 5	55.19%	0.88%	44.03%

Table 11. Distribution of Risk Scores Among Schools Based on the Type of Residential Area
