# Supplementary material for: A quantitative geospatial analysis of the risk that Boko Haram will target a school
Source: PLoS One. 2025 Jun 17;20(6):e0320939. doi: 10.1371/journal.pone.0320939 (PMC12173403; doi:10.1371/journal.pone.0320939)
Supplement: S5 Appendix E — (PDF) [file pone.0320939.s005.pdf]

# Appendix E: Supplementary Tables for the Forest Plots

| | | Estimate | 99% CI, lower | 99% CI, upper | Raw P | FDR |
|---|---|---|---|---|---|---|
| (A) 1km | r=5km | 100.72 | 68.93 | 132.5 | $1.85 \times 10^{-14}$ | $1.85 \times 10^{-14}$ |
| | r=10km | 108.84 | 75.04 | 142.63 | $7.86 \times 10^{-15}$ | $1.05 \times 10^{-14}$ |
| | r=25km | 133.91 | 94.15 | 173.68 | $6.72 \times 10^{-16}$ | $1.34 \times 10^{-15}$ |
| | r=50km | 226.35 | 167.30 | 285.41 | $2.07 \times 10^{-19}$ | $8.29 \times 10^{-19}$ |
| (B) 2km | r=5km | 107.64 | 88.32 | 126.96 | $3.34 \times 10^{-40}$ | $3.34 \times 10^{-40}$ |
| | r=10km | 120.36 | 99.35 | 141.37 | $4.51 \times 10^{-42}$ | $9.03 \times 10^{-42}$ |
| | r=25km | 141.18 | 116.32 | 166.05 | $1.84 \times 10^{-41}$ | $2.45 \times 10^{-41}$ |
| | r=50km | 209.51 | 174.37 | 244.65 | $6.9 \times 10^{-45}$ | $2.76 \times 10^{-44}$ |
| (C) 3km | r=5km | 100.63 | 85.79 | 115.47 | $5.70 \times 10^{-59}$ | $5.70 \times 10^{-59}$ |
| | r=10km | 114.23 | 98.13 | 130.33 | $1.38 \times 10^{-63}$ | $2.77 \times 10^{-63}$ |
| | r=25km | 133.44 | 114.30 | 152.57 | $9.05 \times 10^{-62}$ | $1.21 \times 10^{-61}$ |
| | r=50km | 192.97 | 166.13 | 219.81 | $5.37 \times 10^{-65}$ | $2.15 \times 10^{-64}$ |
| (D) 5km | r=5km | 78.29 | 68.06 | 88.52 | $7.61 \times 10^{-77}$ | $7.61 \times 10^{-77}$ |
| | r=10km | 103.40 | 91.97 | 114.83 | $3.27 \times 10^{-102}$ | $1.31 \times 10^{-101}$ |
| | r=25km | 122.01 | 108.26 | 135.77 | $5.94 \times 10^{-99}$ | $1.19 \times 10^{-98}$ |
| | r=50km | 167.51 | 148.24 | 186.79 | $1.34 \times 10^{-95}$ | $1.78 \times 10^{-95}$ |
| (E) 10km | r=5km | 44.09 | 38.08 | 50.10 | $9.07 \times 10^{-75}$ | $9.07 \times 10^{-75}$ |
| | r=10km | 75.15 | 68.20 | 82.10 | $1.42 \times 10^{-149}$ | $2.83 \times 10^{-149}$ |
| | r=25km | 95.07 | 86.54 | 103.60 | $1.22 \times 10^{-157}$ | $4.89 \times 10^{-157}$ |
| | r=50km | 132.29 | 119.99 | 144.59 | $3.37 \times 10^{-148}$ | $4.49 \times 10^{-148}$ |

**Table 12.** Mean difference estimates in the total number of attacks between Class 1 and Class 0. FDR, false discovery rate*.

*Because the differing km variations are dependent on one another, e.g., all attacks within a 1 km distance are also counted when looking at larger distances, the FDR method that accounts for the correlation structure would be superior to the Bonferroni method.

| | | Estimate | 99% CI, lower | 99% CI, upper | Raw P | FDR |
|---|---|---|---|---|---|---|
| (A) 1km | 1st closest | 28.96 | 19.28 | 38.64 | $3.19 \times 10^{-13}$ | $1.60 \times 10^{-12}$ |
| | 2nd closest | 39.74 | 30.09 | 49.39 | $1.14 \times 10^{-21}$ | $5.70 \times 10^{-21}$ |
| | 3rd closest | 48.20 | 38.99 | 57.42 | $1.06 \times 10^{-30}$ | $5.30 \times 10^{-30}$ |
| | 4th closest | 63.07 | 53.04 | 73.10 | $1.99 \times 10^{-39}$ | $9.94 \times 10^{-39}$ |
| | 5th closest | 71.19 | 59.42 | 82.96 | $1.95 \times 10^{-37}$ | $9.74 \times 10^{-37}$ |
| (B) 2km | 1st closest | 37.54 | 31.79 | 43.28 | $4.64 \times 10^{-52}$ | $2.32 \times 10^{-51}$ |
| | 2nd closest | 41.51 | 35.89 | 47.14 | $5.48 \times 10^{-63}$ | $2.74 \times 10^{-62}$ |
| | 3rd closest | 49.07 | 43.85 | 54.29 | $5.33 \times 10^{-90}$ | $2.66 \times 10^{-89}$ |
| | 4th closest | 62.01 | 56.32 | 67.70 | $8.29 \times 10^{-110}$ | $4.14 \times 10^{-109}$ |
| | 5th closest | 67.31 | 60.74 | 73.88 | $3.64 \times 10^{-101}$ | $1.82 \times 10^{-100}$ |
| (C) 3km | 1st closest | 38.03 | 33.48 | 42.57 | $3.00 \times 10^{-83}$ | $1.50 \times 10^{-82}$ |
| | 2nd closest | 39.79 | 35.34 | 44.24 | $1.06 \times 10^{-92}$ | $5.32 \times 10^{-92}$ |
| | 3rd closest | 46.84 | 42.69 | 50.99 | $1.07 \times 10^{-131}$ | $5.34 \times 10^{-131}$ |
| | 4th closest | 58.86 | 54.28 | 63.44 | $7.24 \times 10^{-158}$ | $3.62 \times 10^{-157}$ |
| | 5th closest | 63.55 | 58.31 | 68.79 | $2.00 \times 10^{-145}$ | $1.00 \times 10^{-144}$ |
| (D) 5km | 1st closest | 37.68 | 34.15 | 41.21 | $1.11 \times 10^{-134}$ | $5.56 \times 10^{-134}$ |
| | 2nd closest | 36.71 | 33.19 | 40.23 | $4.73 \times 10^{-130}$ | $2.37 \times 10^{-129}$ |
| | 3rd closest | 41.88 | 38.57 | 45.19 | $1.78 \times 10^{-176}$ | $8.89 \times 10^{-176}$ |
| | 4th closest | 51.28 | 47.62 | 54.94 | $2.97 \times 10^{-206}$ | $1.49 \times 10^{-205}$ |
| | 5th closest | 54.64 | 50.55 | 58.73 | $2.49 \times 10^{-191}$ | $1.25 \times 10^{-190}$ |
| (E) 10km | 1st closest | 32.59 | 29.95 | 35.24 | $4.39 \times 10^{-188}$ | $2.20 \times 10^{-187}$ |
| | 2nd closest | 30.94 | 28.23 | 33.64 | $2.12 \times 10^{-166}$ | $1.06 \times 10^{-165}$ |
| | 3rd closest | 33.91 | 31.28 | 36.55 | $2.73 \times 10^{-203}$ | $1.37 \times 10^{-202}$ |
| | 4th closest | 40.57 | 37.69 | 43.45 | $4.30 \times 10^{-236}$ | $2.15 \times 10^{-235}$ |
| | 5th closest | 43.80 | 40.61 | 46.99 | $3.99 \times 10^{-226}$ | $2.00 \times 10^{-225}$ |

**Table 13.** Numerical output of statistical analysis of distance to closest security measures.

| | | Estimate | 99% CI, lower | 99% CI, upper | Raw P | FDR |
|---|---|---|---|---|---|---|
| (A) 1km | nga_commun | -0.01 | -0.24 | 0.22 | 0.94 | >0.99 |
| | nga_exposu | 0.13 | 0.09 | 0.17 | $1.56 \times 10^{-14}$ | $4.69 \times 10^{-14}$ |
| | nga_socioe | -0.005 | -0.20 | 0.19 | 0.95 | >0.99 |
| (B) 2km | nga_commun | -0.28 | -0.40 | -0.15 | $2.54 \times 10^{-08}$ | $7.63 \times 10^{-08}$ |
| | nga_exposu | 0.13 | 0.11 | 0.15 | $1.00 \times 10^{-54}$ | $3.01 \times 10^{-54}$ |
| | nga_socioe | -0.33 | -0.46 | -0.21 | $2.84 \times 10^{-12}$ | $8.52 \times 10^{-12}$ |
| (C) 3km | nga_commun | -0.35 | -0.44 | -0.26 | $7.02 \times 10^{-21}$ | $2.11 \times 10^{-20}$ |
| | nga_exposu | 0.13 | 0.11 | 0.14 | $5.71 \times 10^{-92}$ | $1.71 \times 10^{-91}$ |
| | nga_socioe | -0.41 | -0.51 | -0.32 | $4.07 \times 10^{-27}$ | $1.22 \times 10^{-26}$ |
| (D) 5km | nga_commun | -0.44 | -0.51 | -0.38 | $1.45 \times 10^{-60}$ | $4.34 \times 10^{-60}$ |
| | nga_exposu | 0.13 | 0.11 | 0.14 | $2.54 \times 10^{-130}$ | $7.62 \times 10^{-130}$ |
| | nga_socioe | -0.53 | -0.60 | -0.46 | $2.31 \times 10^{-70}$ | $6.92 \times 10^{-70}$ |
| (E) 10km | nga_commun | -0.36 | -0.41 | -0.30 | $2.99 \times 10^{-60}$ | $8.96 \times 10^{-60}$ |
| | nga_exposu | 0.10 | 0.09 | 0.11 | $1.37 \times 10^{-98}$ | $4.10 \times 10^{-98}$ |
| | nga_socioe | -0.45 | -0.51 | -0.39 | $5.43 \times 10^{-77}$ | $1.63 \times 10^{-76}$ |

**Table 14.** Relationship Between the Mean Difference in School Attack Incidences and Socioeconomic Risk Scores Across Various Distances
