# Supplementary material for: A quantitative geospatial analysis of the risk that Boko Haram will target a school
Source: PLoS One. 2025 Jun 17;20(6):e0320939. doi: 10.1371/journal.pone.0320939 (PMC12173403; doi:10.1371/journal.pone.0320939)
Supplement: S6 Appendix F — (PDF) [file pone.0320939.s006.pdf]

Appendix F: OverPass API Queries for Military Installations

0.0.1 Military Installations

```
// Get a CSV output
[out:csv(name,::"type",::"id",::"lat",::"lon";true;",")]
    [timeout:25];

// Limit the search to Nigeria
{{geocodeArea:Nigeria}}->.searchArea;

//Concatenate the results
(
 ["landuse"="military"](area.searchArea);
 relation["landuse"="military"](area.searchArea);
 node["landuse"="military"](area.searchArea);
);

// Print out the results
out center;
>;
```

0.0.2 Military Barracks

```
// Get a CSV output
[out:csv(name,::"type",::"id", ::"lat",::"lon";true;",")]
    [timeout:25];

// Limit the search to Nigeria
{{geocodeArea:Nigeria}}->.searchArea;

// Concatenate the results
(
["military"="barracks"](area.searchArea);
 relation["military"="barracks"](area.searchArea);
 node["military"="barracks"](area.searchArea);
);

// Print out the results
out center;
>;
```

0.0.3 Military Checkpoints

```
[out:csv(name,::"type",::"id",::"lat",::"lon";true;",")]
    [timeout:25];

// Limit the search to Nigeria
{{geocodeArea:Nigeria}}->.searchArea;
```

```
// Concatenate the results
(
 ["military"="checkpoint"](area.searchArea);
 relation["military"="checkpoint"](area.searchArea);
 node["military"="checkpoint"](area.searchArea);
);

// Print out the results
out center;
>;
```

0.0.4 Military Training Area

```
[out:csv(name,::"type",::"id",::"lat",::"lon";true;",")]
    [timeout:25];

// Limit the search to Nigeria
{{geocodeArea:Nigeria}}->.searchArea;

//Concatenate the results
(
["military"="training_area"](area.searchArea);
 relation["military"="training_area"](area.searchArea);
 node["military"="training_area"](area.searchArea);
);

// Print out the results
out center;
>;
```

0.0.5 Military Airfield

```
[out:csv(name,::"type",::"id",::"lat",::"lon";true;",")]
    [timeout:25];

// Limit the search to Nigeria
area[name="Nigeria"];

// Concatenate the results
(
["military"="airfield"](area);
 relation["military"="airfield"](area);
 node["military"="airfield"](area);
);

// Print out the results
out center;
>;
```

0.0.6 Military Naval Base

```
[out:csv(name,::"type",::"id",::"lat",::"lon";true;",")][timeout:25];

// Limit the search to Nigeria
{{geocodeArea:Nigeria}}->.searchArea;

/ Concatenate the results
(
["military"="naval_base"](area.searchArea);
 relation["military"="naval_base"](area.searchArea);
 node["military"="naval_base"](area.searchArea);
);

// Print out the results
out center;
>;
```
