# Supplementary material for: A quantitative geospatial analysis of the risk that Boko Haram will target a school
Source: PLoS One. 2025 Jun 17;20(6):e0320939. doi: 10.1371/journal.pone.0320939 (PMC12173403; doi:10.1371/journal.pone.0320939)
Supplement: S7 Appendix G — (PDF) [file pone.0320939.s007.pdf]

Appendix G: Additional Metrics for ML-Inspired Statistical Analyses

Comparison	PPV	NPV	Sensitivity	Specificity
Urban vs. Rural	0.91911	0.28440	0.03212	0.99270
Urban Centers vs. Clusters	0.89899	0.51417	0.05786	0.99352

Table 15. Urban vs. Rural

Test	PPV	NPV	Sensitivity	Specificity
km=1, test no.1	0.20408	0.99990	0.81081	0.99838
km=1, test no.2	0.20408	0.99997	0.93750	0.99838
km=2, test no.1	0.12255	0.99992	0.89286	0.99503
km=2, test no.2	0.12255	0.99996	0.94340	0.99503
km=2, test no.3	0.29902	0.99965	0.82993	0.99603
km=2, test no.4	0.28922	0.99989	0.93651	0.99597
km=2, test no.5	0.28922	0.99993	0.95935	0.99597

Table 16. Decision Tree Hypotheses
