# Supplementary material for: A quantitative geospatial analysis of the risk that Boko Haram will target a school
Source: PLoS One. 2025 Jun 17;20(6):e0320939. doi: 10.1371/journal.pone.0320939 (PMC12173403; doi:10.1371/journal.pone.0320939)
Supplement: S8 Appendix H — (PDF) [file pone.0320939.s008.pdf]

Appendix H: AdaBoost Confusion Matrices

	**Predicted No**	**Predicted Yes**
**Actual No**	30842	9
**Actual Yes**	26	43

Table 17. Confusion Matrix - k=1

	**Predicted No**	**Predicted Yes**
**Actual No**	30749	12
**Actual Yes**	15	144

Table 18. Confusion Matrix - k=2

	**Predicted No**	**Predicted Yes**
**Actual No**	30653	15
**Actual Yes**	19	233

Table 19. Confusion Matrix - k=3

	**Predicted No**	**Predicted Yes**
**Actual No**	30479	16
**Actual Yes**	13	412

Table 20. Confusion Matrix - k=5

	**Predicted No**	**Predicted Yes**
**Actual No**	30123	9
**Actual Yes**	18	770

Table 21. Confusion Matrix - k=10
