# Supplementary material for: A quantitative geospatial analysis of the risk that Boko Haram will target a school
Source: PLoS One. 2025 Jun 17;20(6):e0320939. doi: 10.1371/journal.pone.0320939 (PMC12173403; doi:10.1371/journal.pone.0320939)
Supplement: S9 Appendix I — (PDF) [file pone.0320939.s009.pdf]

Appendix I: Time-Series Analysis

To analyze the temporal dynamics and evolution of Boko Haram, a time-series dataset covering July 2009 to April 2023 was used. The analysis used a binary classification sequential neural network with two LSTM layers and a dropout layer to prevent overfitting. Despite preprocessing steps such as normalization and cyclical encoding of the months, and splitting the dataset into training (2009-2017), validation (2018-2020), and testing (2021-2023) sets, the model's performance was poor, showing 0% precision, recall, and F1-score across all metrics. This poor performance is a result of the significant data imbalance between the non-conflict and conflict classes—the dataset contained only 76 conflict incidents involving school targets among over 17 million records. As a result, the model predominantly classified records as non-conflict (class 0), as indicated by the confusion matrix.

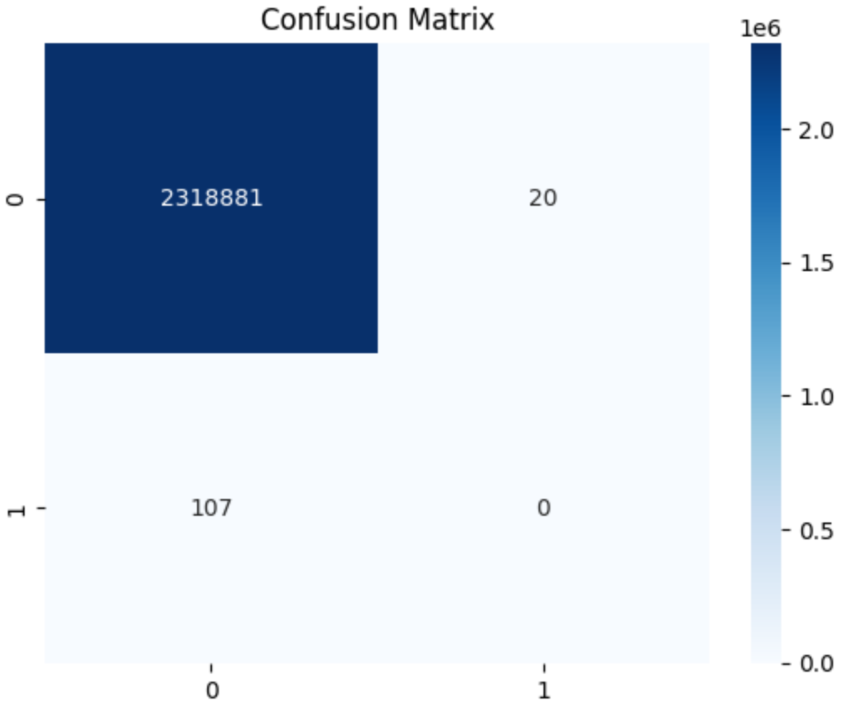

Fig 20. Confusion matrix of running an LSTM on the timeseries version of the dataset.
