# Supplementary material for: A quantitative geospatial analysis of the risk that Boko Haram will target a school
Source: PLoS One. 2025 Jun 17;20(6):e0320939. doi: 10.1371/journal.pone.0320939 (PMC12173403; doi:10.1371/journal.pone.0320939)
Supplement: S10 Appendix J — (PDF) [file pone.0320939.s010.pdf]

Appendix J: Detailed Statistical Methods

For statistical inference, school attacks were chosen as the outcome variable, which we define as "Class 1" (having a school attack) and "Class 0" (not having a school attack). We subsequently quantified the differences in continuous features using linear model-based estimation and discrete features using odds-ratio estimates. Unless otherwise indicated, we computed confidence intervals (CI) and raw P-values of our statistical estimates, followed by accounting for multiple hypothesis testing with an appropriate approach as justified below. All statistical analyses and data visualization were implemented in R 4.0.3 and ggplot2 version 3.5.1, respectively.

Relationship between proxy measure of school attack and outcome classes.

We hypothesized that the total number of any types of attack could be used as a proxy for school attack because this measure might indicate the degree to which Boko Haram was active in a geographical region. To test this hypothesis, we similarly defined "Class 1" (having a school attack) and "Class 0" (no school attack), and compared the mean differences in the total number of attacks between the classes. We used two-sided Welch's t-test for mean difference point estimate, CI, and raw P-value, and visualized the findings with forest plots. We performed the analysis for radii sized 5km, 10km, 25km, and 50km. Because the radii we defined were dependent of each other (i.e., all attacks within a 5km radius were also counted when defining 10km, 25km, and 50km radii from the same center), we applied the false discovery rate (FDR), which considers the correlation structure of the underlying data, to address for multiple hypothesis testing.

Relationship between risk scores and outcome.

We similarly compared each of the risk scores between Class 1 and Class 0. To meet the conditional independence assumption of linear model, we aggregated on distinct schools by summing up all attacks occurred in the given school. To estimate the mean difference between each of the three risk scores (Community, Exposure, and Socioeconomic), we applied a two-sided Welch's t-test, which is a generalization of a linear model. The mean difference in risk scores, defined as the mean score in Class 1 subtracting mean of Class 0, along the CI were computed. The results were summarized and visualized as a forest plot, where the center dot represents an estimate of the mean difference, and the flanking horizontal lines represent the upper and lower bounds of the CI. Raw P-value ¡ 0.05 may be indicated by the horizontal lines not crossing the null value indicated by vertical dotted lines. To address multiple hypothesis testing, the raw P-values were corrected by the Bonferroni method, the most conservative correction approach suitable for any correlation structure. The entire analysis was conducted on 1km, 2km, 3km, 5km, and 10km radii.

Comparison of distance to the nearest security installation.

We hypothesized that there was a rank-ordered, positive association along the security installation in proximity to the location of school attack. To test this, a linear model-based estimation of mean differences between Class 1 and Class 0, implemented as a Welch's t-test, was performed comparing the distance in km between the location of attack to the 1st, 2nd, . . . , 5th nearest known security installation. Mean difference point estimates, CIs, and raw P-values were reported along Bonferroni-adjusted P-values.

Association between outcome classes and population-defined geographical regions.

We quantified the association between the outcome (Class 1 or 0) and geographical density. To this end, a geographical area was assigned "Rural" or "Urban" based on its geographical density ([68]). Odds ratio, representing the enrichment or depeltion of attack within the given geographical classification, was computed with a 2x2 contingency table setup. Statistical uncertainty, including CI and raw P-values, were calculated using the Chi-square with Yates' continuity correction method, which is more suitable for larger sample sizes. Subsequently, we performed another odds ratio analysis restricting to only the "Urban" regions, which could be subdivided into "Urban Centers" and "Urban Clusters" ([68]). Odds ratios, CIs, and P-values were computed in the same manner.

Association of the outcome classes with subdivisions assigned by Decision Tree models.

To determine whether there is a relationship between the outcome classes (1 or 0) and the membership assigned by Decision Tree node-split rules, we similarly implemented the odds ratio analysis. Using the Decision Tree node-split rules described in the main text, a school attack could be assigned Set § or $\bar{S}$. This membership was then subjected to odds ratio, CI, and P-value computation with a 2x2 contingency-table setup and the Chi-square with Yates' continuity correction method as described above.

Additional evaluations of odds ratio analyses.

For analyses involving a 2x2 contingency table-based odds ratio calculation, additional metrics were computed, including sensitivity, specificity, positive and negative predictive values (PPV, NPV), as shown in Table 22.

	**PPV**	**NPV**	**Sensitivity**	**Specificity**
Urban vs. Rural	0.91911	0.28440	0.03212	0.99270
Urban Centers vs. Clusters	0.89899	0.51417	0.05786	0.99352
km=1, test no.1	0.20408	0.99990	0.81081	0.99838
km=1, test no.2	0.20408	0.99997	0.93750	0.99838
km=2, test no.1	0.12255	0.99992	0.89286	0.99503
km=2, test no.2	0.12255	0.99996	0.94340	0.99503
km=2, test no.3	0.29902	0.99965	0.82993	0.99603
km=2, test no.4	0.28922	0.99989	0.93651	0.99597
km=2, test no.5	0.28922	0.99993	0.95935	0.99597

Table 22. Additional metrics for odds ratio analyses.
