# Supplementary material for: A quantitative geospatial analysis of the risk that Boko Haram will target a school
Source: PLoS One. 2025 Jun 17;20(6):e0320939. doi: 10.1371/journal.pone.0320939 (PMC12173403; doi:10.1371/journal.pone.0320939)
Supplement: S11 Appendix K — (PDF) [file pone.0320939.s011.pdf]

Appendix K: Additional Decision Tree - Inspired Statistical Analysis

Hypothesis 7 *Let S be the set of schools that satisfy the following logical condition. Each school $s \in S$ must be such that there are:*

1. *between 7.5 and 80.5 attacks occurred within a 5km radius of the school and*
2. *the number of attacks that occurred within a 25km radius of the school is less than or equal to 17.5 and*
3. *the distance to the nearest security installation to s is less than or equal to 2.73.*

Our hypothesis is that schools in S are far more likely to experience a kidnapping attack by Boko Haram than schools in $\bar{S}$.

As shown in Table 7, the frequency of attacks in S is over 1,600 times ($99\%CI = 685.9 - 4300.3, P = 0.0002$), suggesting that this hypothesis is valid.

Hypothesis 8 *Let S be the set of schools that satisfy the following logical conditions. Each school $s \in S$ must be such that there are:*

1. *between 7.5 and 80.5 attacks occurred within a 5km radius of the school and*
2. *the number of attacks that occurred within a 25km radius of the school is less than or equal to 17.5 and*
3. *the distance to the nearest security installation to s is less than or equal to 2.73km and*
4. *the communication risk score for the school is less or equal to 1.022.*

Our hypothesis is that schools in S are far more likely to experience a kidnapping attack by Boko Haram than schools in $\bar{S}$.

Here, the frequency of attacks in S is approximately 3,200 times higher than $\bar{S}$ ($99\%CI = 1050.3 - 16384.0, P = 0.0002$). Again, this provides statistical evidence that this hypothesis is valid.

Hypothesis 9 *Let S be the set of schools that satisfy the following logical condition. Each school $s \in S$ must be such that there are:*

1. *the number of attacks that occurred within a 5km radius of the school is bigger than 80.5 and*
2. *the socioeconomic risk score for the school is less or equal to 2.283 and*
3. *the number of attacks that occurred within a 25km radius of the school is bigger than 88.5.*

Our hypothesis is that schools in S are far more likely to experience a kidnapping attack by Boko Haram than schools in $\bar{S}$.

Testing suggests that school attacks were over 1,200 times more likely in set S than $\bar{S}$ ($99\%CI = 753.6 - 2015.4, P = 0.0002$, Table 7). Thus, this provides statistical evidence that this hypothesis is valid.

Hypothesis 10 *Let S be the set of schools that satisfy the following logical condition. Each school $s \in S$ must be such that there are:*

1. *the number of attacks occurred within a 5km radius of the school is bigger than 80.5 and*

2. *the socioeconomic risk score for the school is less than or equal to 2.283 and*

3. *the number of attacks occurred within a 25km radius of the school is bigger than 88.5 and*

4. *the distance to the fourth nearest security installation to s is bigger than 116.098km.*

Our hypothesis is that schools in S are far more likely to experience a kidnapping attack by Boko Haram than schools in $\bar{S}$.

Testing revealed over 3,000-fold higher number of attacks in set S than $\bar{S}$ ($99\%CI = 1789.3 - 8192.0, P = 0.0002$, Table 7). Again, this provides statistical evidence that this hypothesis is valid.

Hypothesis 11 *Let S be the set of schools that satisfy the following logical condition. Each school $s \in S$ must be such that there are:*

1. *the number of attacks that occurred within a 5km radius of the school is bigger than 80.5 and*

2. *the socioeconomic risk score for the school is less than or equal to 2.283 and*

3. *the number of attacks that occurred within a 25km radius of the school is bigger than 88.5 and*

4. *the distance to the fourth nearest security installation to s is bigger than 116.098km and*

5. *the number of attacks that occurred within a 10km radius of the school is bigger than 84.5.*

Our hypothesis is that schools in S are far more likely to experience an attack by Boko Haram than schools in $\bar{S}$.

Here, the frequency of attacks is over 5,600 times higher in set S than $\bar{S}$ ($99\%CI = 2409.4 - 16384.0, P = 0.0002$, Table 7).
